# TECPR1 helps bridge the CASM during lysosome damage

**DOI:** 10.15252/embj.2023115210

**Published:** 2023-08-28

**Authors:** Oliver Florey

**Affiliations:** ^1^ Signalling Programme Babraham Institute Cambridge UK

**Keywords:** Membranes & Trafficking, Organelles

## Abstract

Maintaining the integrity of the endolysosomal system is of great importance for cellular homeostasis. Recent work published in *The EMBO Journal* and *EMBO Reports* reveals a novel role for the protein TECPR1 as a sensor for stressed membranes and regulator of lysosomal membrane repair.

## Graphical abstract

TECPR1 acts as cytosolic receptor for exposed sphingomyelin on damaged membranes to regulate non‐canonical autophagy and lysosomal membrane repair.

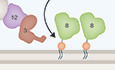

Endolysosomal membrane permeabilization, or their full rupture, is a common and severe stress condition that is relevant for degenerative diseases, infection, cancer, and aging. Regulation of the integrity of the endolysosomal system is important for cellular homeostasis and involves mechanisms whereby cells first sense stressed or damaged membranes and then mount a response to either repair, remove, or replace them. Three new studies now reveal a novel role for the protein TECPR1 as a sensor for stressed membranes, where it plays an unexpected role linking to the autophagy‐associated ATG8 conjugation machinery.

The ATG8 family of ubiquitin‐like proteins, which include LC3A/B/C and GABARAP/L1/L2, play an important role during canonical autophagy. The ATG16L1/ATG5/ATG12 complex acts as an E3 ligase driving the conjugation of ATG8 to the lipid phosphatidylethanolamine in forming autophagosomes (Ichimura *et al*, 2000). Recently, it has been shown that the same ATG8 conjugation machinery also functions in a noncanonical autophagy pathway that involves the conjugation of ATG8 to single membranes (CASM; Durgan & Florey, 2022). CASM is independent of canonical autophagy and occurs at stressed and deacidified endolysosomal compartments. Importantly, there are key molecular differences between CASM and canonical autophagy. First, during CASM, ATG8 proteins can be conjugated to both phosphatidylethanolamine and phosphatidylserine (Durgan *et al*, 2021). Second, ATG16L1 acts as a molecular hub that dictates the location of ATG8 conjugation (Fujita *et al*, 2008), with the central FIP200‐binding domain (FBD) of ATG16L1 being required for its targeting to autophagosome membranes (Gammoh *et al*, 2013; Dooley *et al*, 2014). However, during CASM, ATG16L1 is targeted to endolysosomal membranes via binding of its C‐terminal WD40 domain to the V‐ATPase (Fletcher *et al*, 2018; Hooper *et al*, 2022). In both cases, ATG16L1 was thought to be essential for regulating the site of ATG8 conjugation. However, two studies (Boyle *et al*, 2023; Kaur *et al*, 2023) in The *EMBO Journal* and another in *EMBO Reports* (Corkery *et al*, 2023) challenge this idea and revealed a novel mechanism that activates CASM under conditions of endolysosomal membrane damage (Fig 1).

**Figure 1:**
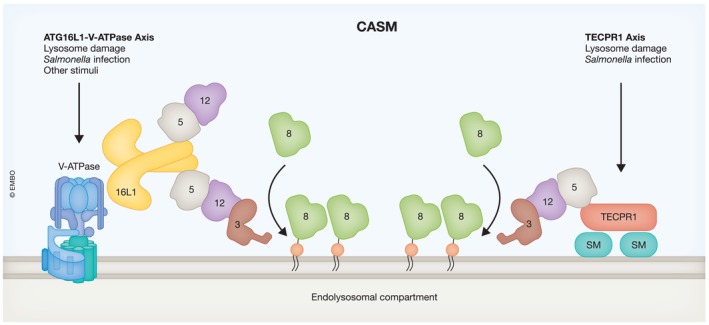
Two molecular mechanisms of CASM activation On the left, the ATG16L1‐V‐ATPase axis and on the right the newly identified TECPR1 axis, where TECPR1 senses and binds sphingomyelin exposed on damaged membranes and recruits ATG5/ATG12 to promote ATG8 conjugation.

The three studies came to same conclusions using different model systems and approaches.

Corkery *et al* and Kaur *et al* both used standard lysosome damaging drugs such as the lysosomotropic agent L‐leucyl‐L‐leucine methyl ester (LLOMe), while Boyle *et al* used a *Salmonella* infection model of endolysosomal membrane damage. All three studies landed on TECPR1 as playing an important novel role in endolysosomal damage. Kaur *et al* started from the striking observation where they detected ATG5‐dependent but ATG16L1‐independent ATG8 conjugation following lysosome damage. This suggested the existence of an alternative ATG5 containing E3 ligase complex. TECPR1 was a clear candidate for this new E3 ligase complex, as it is a known ATG5 interacting partner (Chen *et al*, 2012). Indeed, while single knockout of either ATG16L1 or TECPR1 was not able to block ATG8 conjugation, knockout of both proteins fully blocked LLOMe‐induced ATG8 conjugation. Corkery *et al* started with TECPR1 as a potential candidate protein involved in lysosome damage. The team observed robust recruitment of TECPR1 to damaged lysosomes and observed the same TECPR1‐dependent residual ATG8 conjugation in ATG16L1 knockout cells following lysosome damage. Importantly, in agreement with Kaur *et al*, this ATG8 conjugation was independent of canonical autophagy and instead represented CASM.

A key question raised by these findings was how TECPR1 was targeted to damaged endolysosomal membranes, which was where the study by Boyle *et al* began. It was known that *Salmonella*‐induced membrane damage resulted in the cytosolic exposure of sphingomyelin (SM; Ellison *et al*, 2020). However, how SM exposure is sensed by the host cell was unknown. Using mass spectrometry analysis of liposome‐based assays mixed with cell lysates, Boyle *et al* identified TECPR1 as a SM‐binding protein. In agreement with the other studies, Boyle *et al* also demonstrated a role for TECPR1 in supporting ATG8 conjugation to *Salmonella* containing vacuoles in an ATG16L1‐independent manner. In reciprocal agreement, both Corkery *et al* and Kaur *et al* also. demonstrated SM exposure during lysosome damage, which was required for TECPR1 recruitment and ATG8 conjugation. All three groups went on to identify the N‐terminal dysferlin domain of TECPR1 as being essential for SM binding, with Kaur *et al* and Boyle *et al* further providing crystal structures of the interaction and identifying critical residues within the domain.

Interestingly, while lysosome damage and *Salmonella* infection‐induced CASM led to SM exposure and TECPR1‐dependent ATG8 conjugation, other stimuli of CASM, such as the ionophore monensin, did not. Thus, there appears to be differential molecular mechanisms for CASM activation that are dependent on the stimuli. In terms of pathogen infection, this makes some evolutionary sense, as it was recently shown that the *Salmonella* effector protein SopF can inhibit the ATG16L1‐V‐ATPase axis of CASM activation (Ulferts *et al*, 2021; Hooper *et al*, 2022). Therefore, the development of an ATG5‐TECPR1 axis could act as a backup mechanism to activate CASM in host cells. Questions still remain as to the function of CASM during lysosome damage, with Corkery *et al* presenting data that suggest a role for TECPR1‐mediated CASM in lysosome repair. Boyle *et al* suggest that high levels of TECPR1 recruitment to *Salmonella* containing vacuoles accentuate their damage, which may result in a beneficial host response to clear the pathogen by macroautophagy.

These studies elegantly reveal TECPR1 as a new E3 ligase of ATG8 conjugation and a sensor of SM exposure, relevant to CASM induced by membrane damage. These exciting findings greatly expand our molecular understanding of CASM. While the evidence still points to the majority of CASM as being mediated by the ATG16L1‐V‐ATPase axis, it is now clear that there are alternative mechanisms at play, which are stimuli specific and may have cell type‐specific contexts.
